# A whole-genome RNAi screen uncovers a novel role for human potassium channels in cell killing by the parasite *Entamoeba histolytica*

**DOI:** 10.1038/srep13613

**Published:** 2015-09-08

**Authors:** Chelsea Marie, Hans P. Verkerke, Dan Theodorescu, William A. Petri

**Affiliations:** 1 Division of Infectious Diseases and International Health, University of Virginia School of Medicine, Charlottesville, Virginia USA; 2 Department of Surgery, Department of Pharmacology, University of Colorado Comprehensive Cancer Center, University of Colorado, Denver, CO, USA

## Abstract

The parasite *Entamoeba histolytica* kills human cells resulting in ulceration, inflammation and invasion of the colonic epithelium. We used the cytotoxic properties of ameba to select a genome-wide RNAi library to reveal novel host factors that control susceptibility to amebic killing. We identified 281 candidate susceptibility genes and bioinformatics analyses revealed that ion transporters were significantly enriched among susceptibility genes. Potassium (K^+^) channels were the most common transporter identified. Their importance was further supported by colon biopsy of humans with amebiasis that demonstrated suppressed K^+^ channel expression. Inhibition of human K^+^ channels by genetic silencing, pharmacologic inhibitors and with excess K^+^ protected diverse cell types from *E. histolytica-*induced death. Contact with *E. histolytica* parasites triggered K^+^ channel activation and K^+^ efflux by intestinal epithelial cells, which preceded cell killing. Specific inhibition of Ca^2+^-dependent K^+^ channels was highly effective in preventing amebic cytotoxicity in intestinal epithelial cells and macrophages. Blockade of K^+^ efflux also inhibited caspase-1 activation, IL-1β secretion and pyroptotic death in THP-1 macrophages. We concluded that K^+^ channels are host mediators of amebic cytotoxicity in multiple cells types and of inflammasome activation in macrophages.

*E. histolytica* is a major cause of severe diarrhea globally^1^,^2^,^3^. Amebiasis has a global distribution of more than 50 million cases worldwide, with an estimated 40,000–110,000 deaths and there are limited effective therapeutic options. For invasive amebiasis the nitroimidazoles are the only approved drug class, for which toxicity and the emergence of resistance are clinical concerns. In Dhaka, Bangladesh 45% of infants were infected with *E. histolytica* and 11% suffered from *E. histolytica* diarrhea in their first year of life^4^. *E. histolytica* was a leading cause of unadjusted mortality from 12 to 24 months of age in a 7-site study of moderate to severe diarrhea in low income countries^1^, and has been associated with growth shortfall and impaired cognitive development^5^,^6^,^7^. Amebiasis causes significant global morbidity, and unacceptably remains a cause of mortality in children in the developing world.

The name is derived from its potent cytotoxic activity toward host cells—*histolytica-* is a composite of Greek roots meaning tissue-loosening. Detailed analysis of *E. histolytica* killing of host cells has uncovered a distinctive cytopathic mechanism, termed trogocytosis (nibbling)^8^. In trogocytosis, *E. histolytica* trophozoites attach to and internalize pieces of the host cell membrane, leading to Ca^2+^ elevations and rapid death of the target cells^8^. Killed cell can trigger a potent inflammatory immune response leading to macrophage and neutrophil infiltrates^9^ and allow parasite invasion of colonic crypts. Parasites also induce host inflammatory signaling cascades at the molecular level via activation of extracellular regulated kinases 1 and 2 and NADPH-oxidase-derived reactive oxygen species production^10^,^11^,^12^,^13^.

Clinical studies have shown that host inflammatory mediators including leptin^14^, tumor necrosis factor-α^15^, and interferon-γ^16^ can strongly influence amebic pathogenesis. Other host molecules implicated in amebic pathogenesis at the cellular level include the apoptosis-regulator Bcl2 and the transcriptional regulators NF-κB and Stat3^17^,^18^. In combination these studies demonstrate the importance of host factors for the outcome of amebic infection.

In order to identify novel and biologically relevant host factors required for amebic cytotoxicity, we selected a whole genome pooled RNAi library of human cells for resistance to amebic killing. This approach has been used successfully to identify host factors that mediate susceptibility to viral and bacterial pathogens and recently for the parasite *Trypansoma cruzi*^19^. We hypothesized that cells silenced for host factors that are exploited by *E. histolytica* would exhibit increased survival to killing by *E. histolytica* parasites.

Our RNAi screen identified a novel and important role for ion transport for host cell resistance to amebic killing. Many enteric infections lead to dysregulation of host ion transport, and reduced absorption and increased secretion at the intestinal lumen results in diarrhea^20^,^21^,^22^. The role of host ion transport in the pathogenesis of *E. histolytica* at the intestinal epithelium is relatively unexplored. Early work described that amebic lysates inhibited colonic Na^+^ and Cl^−^ absorption and stimulated Cl^−^ secretion in rat colonic tissue^23^,^24^. Cl^−^ secretion was mediated by a Ca^2+^-dependent response activated by amebic serotonin^24^ and by cAMP activation of the cystic fibrosis conductance regulator (Cftr)^23^. *E. histolytica* analogs of serotonin and prostaglandin E2 have been shown to induce increased intracellular cAMP and Ca^2+^ upstream of host inflammatory and secretory responses^25^,^26^.

K^+^ channels were identified in the RNAi screen and were uncharacterized in amebiasis. We further explored the role of K^+^ channels as mediators of cell death by *E. histolytica*. We found that inhibition of host K^+^ efflux blocked amebic killing of multiple cell types including intestinal epithelial cells and macrophages. In addition, *E. histolytica* activated host K^+^ channels in human cells upon contact and inhibitor studies indicated a primary role for Ca^2+^-dependent K^+^ channels. K^+^ efflux was necessary for *E. histolytica* activation of caspase-1 and inflammasome-mediated secretion of IL-1β in human macrophages. These results demonstrate that *E. histolytica* parasites actively modify cellular ion transport resulting in ionic secretion, activation of an inflammatory cascade in some cell types, and cell death. Here we report the methodology and results of the RNAi screen, the analysis and validation of RNAi candidate genes and characterization of K^+^ transport as a critical mediator of amebic cytotoxicity.

## Results

### Design and implementation of a whole genome shRNA screen to identify novel host factors in *E. histolytica* cytotoxicity

We directly select a pooled genome-wide RNAi library for clones with increased resistance to killing by *E. histolytica* parasites. The library was constructed in UMUC3 cells, which were susceptible to killing by *E. histolytica*. In addition UMUC3 killing was blocked by galactose, which blocks amebic adherence and contact-dependent killing (Fig. S1A). To define optimal screening conditions, we tested the effect of cell density and host cell: parasite ratio. We selected a ratio of 1:5 parasites to host cells, which yielded ~22% killing of host cells after 3 hours of contact with lower density plated library cells (Fig. S1B).

**Figure 1:**
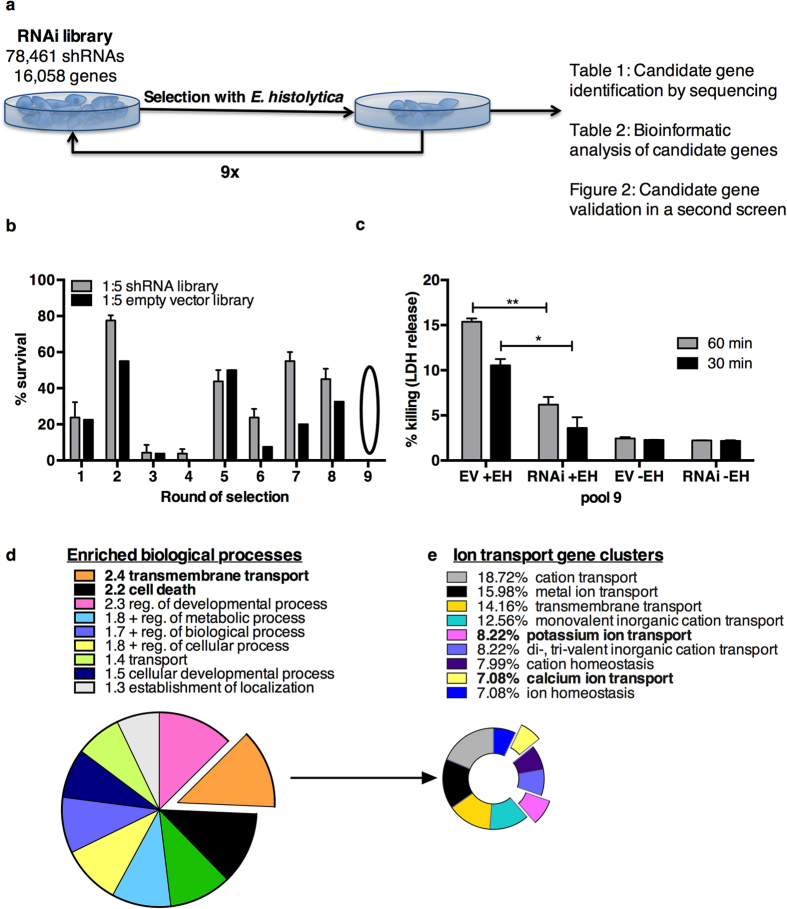
Design and implementation of a whole genome RNAi screen to identify host factors required for amebic cytotoxicity(**a**) Screening of the RNAi library with *E. histolytica* parasites. After each selection, the parasites were removed and resistant cells were expanded and selected successively at a ratio of 1 parasite to 5 host cells, except for the ninth and final round of selection, (1 parasite to 2 host cells). (**b**) Selection increased resistance to amebic killing. Pool 6 of the RNAi library pool exhibited increased resistance relative to an empty vector pool selected in parallel after 6 rounds of selection. In the ninth and final round of selection each library was selected until no surviving cells were visible by microscopic examination. Percent survival of UMUC3 cells was determined by visual assessment of the monolayers after selection. Cells that survived round 9 of selection (pool 9) were sequenced to identify candidate genes. (**c**) The selected RNAi library (RNAi) and the empty vector (EV) control from pool 9 of the screen were assayed for resistance to amebic killing. The mean of biological triplicates and s.e.m. is shown and analyzed by 2-tailed t test (*P < 0.01, **P < 0.001) (**d**) Bioinformatics analysis of candidate susceptibility genes. The top overrepresented biological processes are shown with their enrichment value. (**e**) Clusters of ion transport associated processes in candidate genes. Clusters were based on similarity (kappa statistic >0.3). The percent of genes in each cluster is shown.

The input pooled shRNA library was subjected to successive rounds of selection with *E. histolytica* trophozoites. After each round of selection, resistant cells were separated from trophozoites and cultured to obtain a sufficient cell number for rescreening. Samples were taken after every round of selection to track the loss of susceptible clones (Fig. 1a). The RNAi library had increased resistance to *E. histolytica* cytotoxicity relative to the empty vector control library screened in parallel after 6 rounds of selection (Fig. 1b). The screen was continued for three additional rounds of selection, with the final round of selection at the higher ratio of 1:2 parasites to host cells.

### Candidate susceptibility gene identification by next generation sequencing

DNA from resistant clone pools was purified and sequenced by next generation sequencing. The number of sequence reads corresponding to a given shRNA construct was used to estimate the relative abundance of individual clones. The sequence reads displayed a normal distribution in all the sequenced pools. Clones with fewer than 10 sequencing reads were excluded from this analysis (Fig. S1C). Pool six contained 5320 unique TRCN clones targeting 4314 genes, representing ~27% of the genes in the input library. This was reduced to 410 TRCNs targeting 395 genes in pool 8 and further to 284 TRCNs targeting 281 genes in pool 9 (Table 1). The low number of clones lost between round 8 and round 9 was consistent with saturation of the selection by *E. histolytica*. A full list of TRCN clone IDs, sequence abundance, and corresponding gene targets is provided in Table S1.

### Bioinformatics Analysis of Resistant Clones

We used gene set enrichment analysis to classify the susceptibility candidate genes into statistically significant over-represented functional categories (Table S3). Several overrepresented gene categories with previously documented roles in amebic cytotoxicity included cell death (25 genes, fold enrichment = 2.2*, P* = 0.0004) and calcium signaling (9 genes, KEGG, 3.5 fold enrichment*, P* = 0.0035) were identified (Tables 2 and S3). Genes in pathways with previously defined roles in *E. histolytica* cytotoxicity, included: sugar modifying enzymes (Alg1, Alg9, B3gnt7, Ogt, Gbe1, PigV, Pgk2, Gp6)^27^,^28^, fibronectin genes (Fndcb3, Flrt3)^29^,^30^, caspase-8 (Casp8)^31^, Cftr^24^, protein phosphatases (Acp1, Ppp1r13l, Ppp1r14c, Ppp2r1b, Ppp3r1, Ppp4r1l)^32^ and Ca^2+^ binding proteins (Cib3, Cabp2, Cacng8, Scgn, Slc24a3)^8^,^33^,^34^. The selection of previously discovered genes in the RNAi screen indicated this approach selected for biologically relevant susceptibility genes.

Ion transmembrane transport stood out as significantly overrepresented molecular function (MF) (2.2-fold enriched, P = 0.00065) in the candidate gene set. 24 genes were classified as ion transporters, with 18 cation transporters (2.0 fold enriched, P = 0.01). The predominant substrate was K^+^ (5 genes), followed by Cl^−^ (4 genes including Cftr), Na^+^ (3 genes) and Ca^2+^ (2 genes) (Fig. 1d). Transmembrane transport was also the most enriched biological process (BP) by gene ontology analysis, (2.4-fold enrichment, P = 0.0006). The transport-related biological processes that were enriched in the candidate genes are shown in Fig. 1d. Cellular component (CC) analysis localized 8 candidate genes to ion channel complexes (2.6-fold enrichment, *P* = 0.03) as was expected based on MF and BP enrichment (Table 2). Thus ion transport was significantly overrepresented as a molecular function, biological process and cellular component in candidate susceptibility gene set (Table 2, Fig. 1d). This analysis furthermore suggested a novel role for K^+^ ion transport in ameba-induced cell death (Table 3).

### Evidence score analysis of candidate susceptibility genes

We combined several available datasets to systematically prioritize the 281 candidate genes to generate an evidence score, a technique described previously for candidate gene analysis in a whole genome screen^35^. The evidence score incorporated the relative survival of individual clones (determined by sequence reads) over the course of the screen as well as selection with multiple shRNA constructs (3 genes). Other criteria included: colonic expression in the EST database because the colon is the main site of *E. histolytica* infection (61 genes)^36^, annotation in the KEGG amebiasis pathway (hsa05146) (19 genes) and differential regulation during *E. histolytica* infection (8 genes)^37^,^38^. Two transcriptional analyses were used to identify significant differentially regulated genes: one compared colonic gene expression in patients with acute amebic colitis to matched convalescent colonic biopsies^39^, and the other measured induction in response to *E. histolytica* in host cells *in vitro*^40^. The criteria and genes with evidence scores are provided in Table S2. Fibronectin type III domain containing 3B (Fndc3b) had the highest evidence score of 8 (Table S2). The fibronectin leucine rich transmembrane protein 3 (Flrt3) had an evidence score of 4. The prioritization of these genes validated this approach as *E. histolytica* is known to recognize and bind host fibronectin^29^,^30^.

### Validation of selected candidate susceptibility genes in a secondary RNAi screen

**Figure 2:**
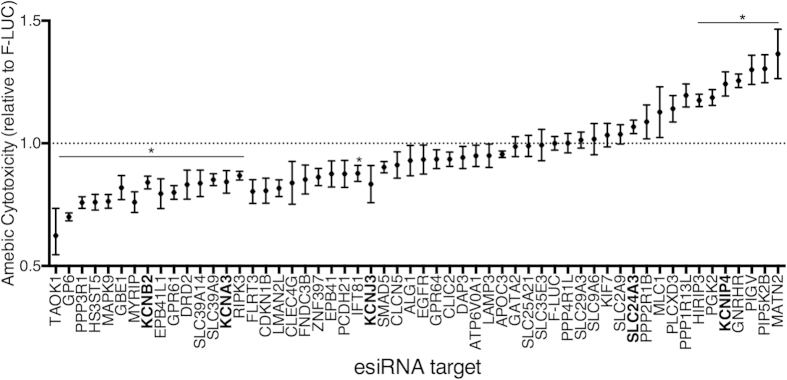
Validation of candidate susceptibility genes in a secondary screen. 55 candidate genes were selected for validation by esiRNA. Cytotoxicity was normalized to FLUC controls for each knockdown. K^+^ channels are bolded. The means of triplicate experimental values were averaged for three independent experiments. The error bars represent the range of means in triplicate independent experiments. *P < 0.01 calculated for candidate genes relative to FLUC by one-way ANOVA Fisher’s LSD test (each comparison to FLUC stands alone).

Due to the high probability of off target effects in pooled shRNA screens we tested 55 candidate susceptibility genes in a secondary screen. In this screen each candidate gene was silenced with endoribonuclease-digested siRNA (esiRNA) and amebic cytotoxicity was directly measured. 35 of 55 candidate susceptibility genes reduced amebic cytotoxicity relative to the FLUC control. 15 reached significance in triplicate assays (1-way ANOVA, P < 0.05). 9 knockdowns had marginal effects on amebic killing (<5%) and 12 knockdowns increased susceptibility to amebic cytotoxicity, 7 of which reached significance (1-way ANOVA, P < 0.05) (Fig. 2). Overall, 65% of hits from the primary screen reduced amebic cytotoxicity in the secondary screen, including Flrt3, the gene with the highest evidence score. Flrt3 knockdown reduced amebic killing by 20% relative to controls. It is of note that K^+^ ion channels that were not validated (Kcnip4 and Slc24a3) had evidence scores of <2, while the K^+^ channels with higher evidence scores (Kcnb2, Kcna3, Kcnj3) significantly reduced amebic killing by ~16% (*P* < 0.05) (Fig. 2 and Table 3).

### Inhibition of ion transport blocked amebic cytotoxicity

**Figure 3:**
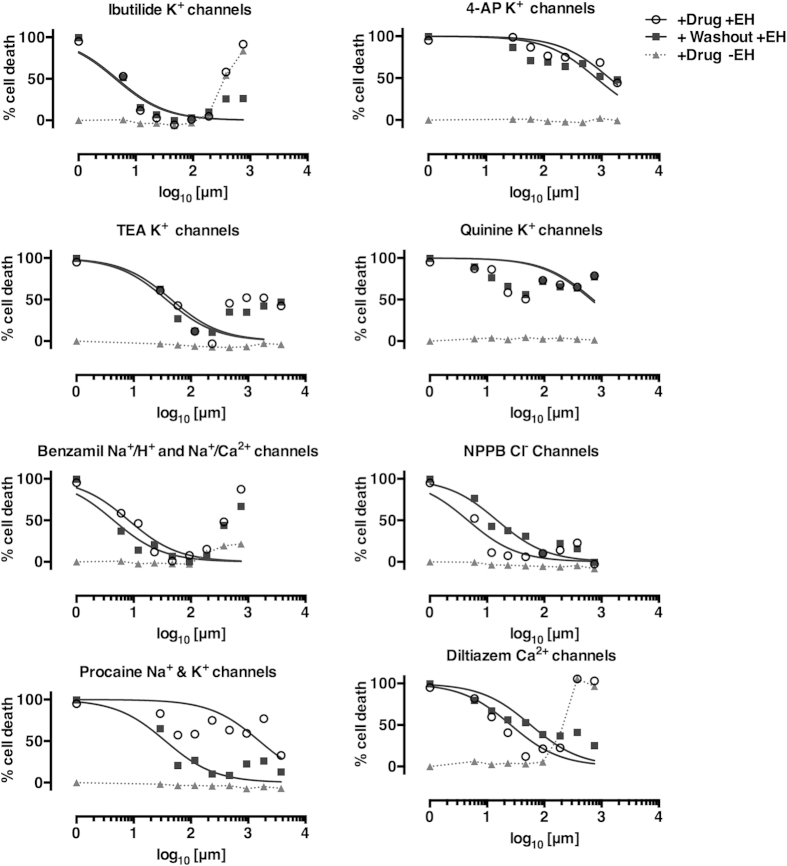
Specific ion transport inhibitors blocked amebic cytotoxicity in UMUC3 cells. Inhibitors of ion channels hits from the RNAi screen were tested for the ability to block amebic cytotoxicity *in vitro* in UMUC3 cells. Cells were treated for 30 minutes at concentrations shown. We tested washout of inhibitors (+Washout +EH) relative to no washout(+Drug +EH). *E. histolytica* trophozoites were added at a ratio of 1:5 trophozoites to host cells. The addition of *E. histolytica* resulted in a 1:2 dilution of the inhibitors (concentrations prior to dilution are shown). LDH release was measured after 30 minutes. IC_50_ was determined by non-linear regression of the log_10_ of the inhibitor concentration vs. normalized cytotoxicity. IC_50_ determinations excluded drug concentrations that resulted in toxicity to UMUC3 cells in the absence of *E. histolytica.*

To further validate the role of ion transport in amebic cytotoxicity, we tested several known pharmacological inhibitors in UMUC3 cells. The broad-spectrum K^+^ channel inhibitors ibutilide (IBU) and tetraethylammonium chloride (TEA) inhibited amebic cytotoxicity to an undetectable level (Fig. 3). Ibutilide inhibits both K^+^ and Ca^2+^ channels and blocked amebic killing with an IC_50_ of ~5 μM, nearly 50-fold higher than the reported IC_50_ for specific inhibition of rapid component of the cardiac delayed rectifier potassium current K^+^ channels (0.01–2 μM)^39^,^40^,^41^,^42^. TEA blocks a range of K^+^ channels at different concentrations including: Ca^2+^-activated K^+^ channels (IC_50_: 150 μM), delayed-rectifier K^+^ channels (IC_50_ = 3 mM) and ATP-activated K^+^ channels (IC_50_: 15 mM)^43^. The IC_50_ of TEA for inhibition of amebic cytotoxicity was ~50 μM, consistent with specific inhibition of Ca^2+^-activated K^+^ channels. TEA and IBU were toxic to cells in the absence of ameba at high concentrations (>1 mM IBU, >2.34 mM TEA).

Two other broad broad-spectrum K^+^ channel inhibitors, Quinine and 4-aminopyrimadine (4-AP) decreased amebic cytotoxicity marginally (Fig. 3). 4-AP caused toxicity above 18.75 mM while quinine did not cause cytotoxicity at the concentrations tested. Quinine is an antimalarial compound with similar inhibitory effects as TEA, but less efficacy against Ca^2+^-activated K^+^ channels (IC_50_: 300 μM)^43^.

Other ion channel inhibitors also blocked amebic killing of UMUC3 cells. The K^+^/Na^+^/Ca^2+^ channel inhibitor benzamil inhibited amebic cytotoxicity with an IC_50_ of ~8 μM consistent with other studies^44^,^45^. The anesthetic procaine blocked amebic cytotoxicity with an IC_50_ 36 μM. Procaine has reported effects on Na^+^ and K^+^ channels (IC_50_ for Na^+^ channels: 110 μM, voltage-activated K^+^ channels: 6302 μM, inward-rectifying K^+^ channels: 35 μM)^46^. The Ca^2+^ channel inhibitor diltiazem is active in the colon^47^ and has a range of concentration-dependent activities (0.5–2500 μM)^48^. Diltiazem blocked amebic cytotoxicity with an IC_50_ 25–60 μM, consistent with inhibition of Ca^2+^ channels^48^. The Cl^−^ channel blocker 5-nitro-2-(3-phenylpropylamino)benzoic acid (NPPB) blocked amebic cytotoxicity with an IC_50_ 5–15 μM (Fig. 3) consistent with the reported IC_50_ for Cl^−^ channels in intestinal T84 cells (20 μM)^99^. Overall, the finding that pharmacological ion channel inhibitors strongly blocked amebic killing supported a role for the importance of ion flux as a primary mechanism in amebic cell killing. Data are summarized in Table 4.

### Colonic gene regulation in human amebic colitis

**Figure 4:**
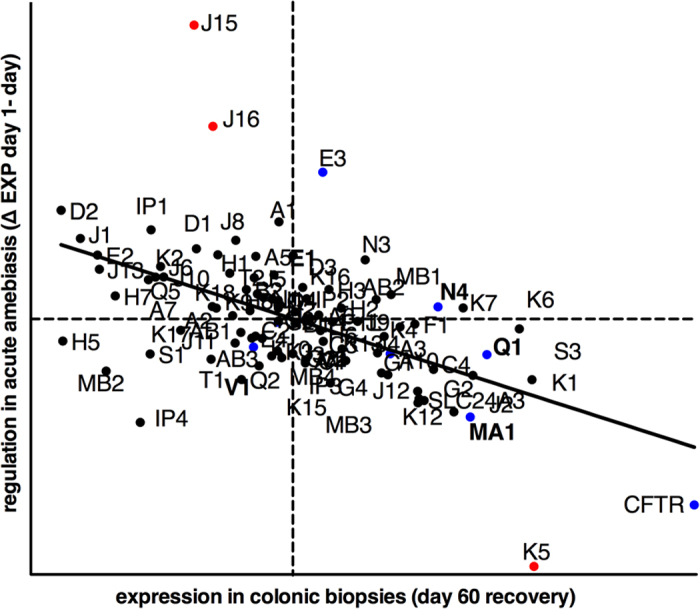
Kcn gene expression and regulation in the human colon in amebiasis. Average gene expression (day 60) and regulation during acute amebiasis (Δ day 1–day 60) of the 94 annotated Kcn genes. Kcn was omitted from K^+^ channel gene names for clarity. Slc24a3 and Cftr are shown for reference. Kcn genes with documented intestinal expression are in blue. Genes that were significantly differentially regulated in disease are shown in red. Significance was determined as P < 0.001 by 2-way ANOVA of matched day 1 and day 60 samples (n = 8). Quadrants indicated the mean of expression (day 60) and regulation (Δ day 1–day 60) of all probes on the microarray. Normal expression (day 60) was significantly correlated with the regulation during disease (w day 1–day 60) by linear regression (n = 8 matched samples, R = −0.54 P > 0.0001.

The primary RNAi screen identified ion channels as mediators of cell killing in the bladder epithelial cell line UMUC3. Ion channels and their regulatory subunits exhibit tissue specific expression^50^. To establish the role of ion transport in colonic amebic infection we examined the expression of K^+^ channel (Kcn) genes during *E. histolytica* infection of the human colon^37^ (Fig. 4). We compared expression of the 94 annotated *Kcn* genes during acute amebic colitis in humans to matched biopsies from recovered patients. *Kcn* genes were globally down-regulated during acute amebiasis (day 1 of acute amebiasis). Furthermore, the level of down-regulation correlated with the degree of expression in the colon in recovered patients (60 days post infection expression) (R = −0.54, *P* < 0. 0001). The Cl^−^ channel Cftr was also highly expressed in the colon and was down-regulated during acute amebiasis. Down-regulation of colonic Kcn genes and Cftr may be a protective physiological response to prevent excessive luminal secretion and cell death during amebiasis. Additionally, cells with higher Kcn gene expression may be more sensitive to *E. histolytica* killing leading to an overall depletion of Kcn transcripts in the colon during amebic infection.

### Inhibition of ion channels blocked amebic killing of intestinal epithelial cells and macrophages

**Figure 5:**
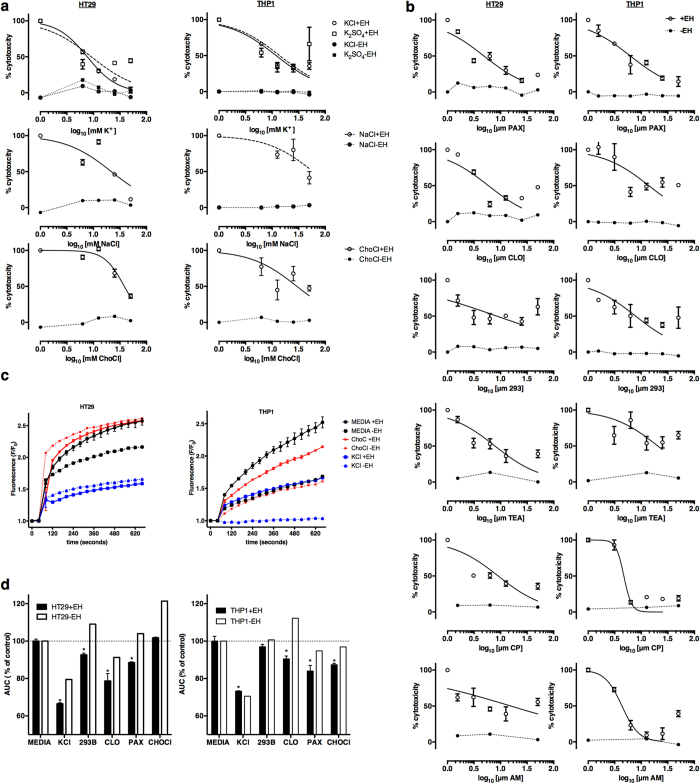
K^+^ inhibitors blocked amebic cytotoxicity and K^+^ activation by *E. histolytica* in intestinal epithelial cells and macrophages. (**a**) Chemical inhibition of ion transport blocked amebic cytotoxicity. Cells were switched to the indicated concentrations of chemicals immediately prior to the addition of *E. histolytica.* (**b**) Specific K^+^ channel inhibitors blocked amebic cytotoxicity. Cells were treated with inhibitors for 30 minutes prior to the addition of *E. histolytica*. (**c**) K^+^ channel activation by *E. histolytica.* Fluorescence values correspond to thallium influx through open K^+^ channels. F values were normalized to the initial baseline value. *E. histolytica* (+EH) or vehicle (−EH) was added after 40 seconds. The mean of 3 biological replicates and the range is shown (+EH), −EH values are single measurements. (**d**) Inhibitors blocked K^+^ channel activation by *E. histolytica.* The mean of the area under the curve (AUC) for inhibitor-treated cells (KCl and ChoCl: 25 mM, 293B, CLO, PAX: 10 μ0) with (+EH) and without *E. histolytica* (−EH) is shown. The AUC (% of control) for each inhibitor was normalized to the media control (+EH was normalized to +EH control, −EH was normalized to −EH control). (+EH is the AUC of three biological replicates; error bars represent the range of measurements. *P < 0.001 calculated for each inhibitor relative to untreated cells by Fisher’s LSD test.

To further define the functional classes of ion transporters in physiologically relevant intestinal and immune cells we tested the ability of increased extracellular ions and specific pharmacological inhibitors to block amebic killing of HT-29 intestinal epithelial cells (IECs) and THP-1 macrophages. High extracellular KCl and K_2_SO_4_ blocked amebic cytotoxicity in IECs and macrophages. Extracellular KCl blocked killing with an IC_50_ of 7.64 mM in IECs and 11.9 mM in macrophages (Fig. 5a). K_2_SO_4_ showed similar inhibition of killing as KCl, indicating that the ionic effects are due to K^+^ rather than Cl^−^ ions. NaCl blocked amebic cytotoxicity at higher concentrations (IEC IC_50_ = 21.8 mM , macrophage IC_50_ = 58.9 mM). Choline chloride was included as an osmotic control and inhibited cytotoxicity in macrophages (IC_50_ = 29.2 mM) and IECs (IC_50_ = 38.1 mM). Both NaCl and ChoCl at high concentrations can modulate K^+^ channels including Kcnma1, that are regulated by extracellular Na^+^ and Cl^−^ ion concentrations^51^.

### Specific ion channels inhibitors blocked amebic killing of intestinal epithelial cells and macrophages

*E. histolytica* triggers a rapid increase intracellular Ca^2+^^8^,^33^ and cAMP^23^ in host cells, both of which can activate K^+^ channels. We tested the efficacy of specific Ca^2+^-activated K^+^ channel inhibitors (paxilline and clotrimazole) and the specific inhibitor of cAMP-activated K^+^ channels (chromanol 293B) for blocking amebic cytotoxicity. Paxilline inhibits big conductance (BK) Ca^2+^-activated K^+^ channels, mainly Kcnma1 in the apical membrane of goblet cells in the intestine^49^. Paxilline blocked amebic killing of IECs (84± 3% reduction, IC_50_ = 4.86 μM) and of macrophages (85± 7% reduction, IC_50_ = 6.19 μM). Paxilline has a higher affinity for the closed conformation of KcnMA1 (IC_50_ = 10 nM) relative to the open conformation (IC_50_ = 10 μM)^52^,^53^. The IC_50_ of paxilline for amebic killing is consistent with inhibition of the open conformation of KcnMA1 (Fig. 5b and Table 5). Clotrimazole is a potent inhibitor of intermediate-conductance (IK) Ca^2+^-activated K^+^ channels. Clotrimazole reduced amebic killing of IECs (76± 4% reduction, IC_50_ = 5.90 μM) and macrophages (60± 6%, IC_50_ = 13.9 μM) (Fig. 5b and Table 5). The major target of clotrimazole in the intestine is KcnN4 which is also activated by intracellular cAMP in parallel with Cftr to drive intestinal CI^−^ secretion^54^.

In the intestine, Kcnq1 forms a voltage-insensitive channel with the Kcne1 subunit that is activated by elevated intracellular cAMP and may be co-activated with Cftr^54^,^55^,^56^,^57^. The specific inhibitor of Kcnq1, chromanol 293B, reduced amebic killing of IECs (57± 5%, IC_50_ = 7 μM) and macrophages (62± 3%, IC_50_ = 7 μM) (Fig. 5b and Table 5). Overall, these data show that inhibition of multiple classes of K^+^ channels blocked amebic killing of intestinal epithelial cells and macrophages at concentrations reported to block K^+^ current in electrophysiological studies^58^,^59^,^60^.

Paxilline was the most effective inhibitor in both IECs and macrophages, and the paxilline target, Kcnma1, was the third most down-regulated Kcn gene during acute amebic colitis. Further the K^+^ channels targeted by paxilline (Kcnma1), clotrimazole (Kcnn4) and chromanol 293b (Kcnq1) were highly expressed in colonic biopsies (Fig. 4). To further define the mechanism of *E. histolytica* activation of K^+^ channels in the intestine we tested specific K^+^ channel inhibitors of K^+^ channels identified in the RNAi screen in IECs and macrophages. AM 92016 and CP 339818 are potent blockers of voltage-gated K^+^ channels, mainly KcnA3 and KcnA2^61^,^62^, while TEA is a broad inhibitor of K^+^ channel activity^63^. AM 92016 and CP 339818 blocked killing of IECs and macrophages but more effective in macrophages from amebic killing (Fig. 5b and Table 5).

### *E. histolytica* trophozoites activated K^+^ channels in IECs and macrophages

We tested of *E. histolytica* induced K^+^ channel activation with the FluxOR assay. We found that increased extracellular K^+^ blocked amebic K^+^ channel activation in IECs and macrophages. Similar to the effect on cell killing, choline chloride had no effect on K^+^ channel activation in IECs but moderately reduced K^+^ channel activation in THP-1 macrophages (Fig. 5c). To test if inhibitors specifically inhibited K^+^ channel activation by *E. histolytica* we compared channel activation in the presence and absence of trophozoites (Fig. 5d). KCl, paxilline, clotrimazole and chromanol 293B reduced K^+^ channel activation in HT-29 cells in response to *E. histolytica*. It is of note that KCl and clotrimazole also reduced K^+^ channel activation in the absence of *E. histolytica.* A similar response was observed in THP-1 macrophages, except that chromanol 293B did not block K^+^ channel activation by *E. histolytica* and choline chloride only moderately reduced K^+^ channel activation in response to *E. histolytica* (Fig. 5d).

### *E. histolytica* trophozoites caused K^+^ efflux in IECs

**Figure 6:**
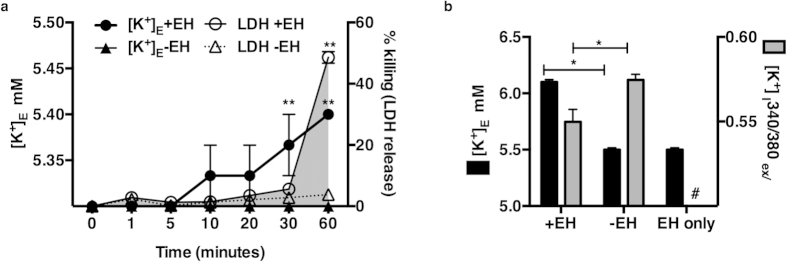
*E. histolytica* induced K^+^ efflux in IECs. (**a**) Extracellular K^+^ ([K^+^ mM]_E_) concentrations were measured in supernatants of HT-29 cells incubated with or without *E. histolytica* at a ratio of 1 trophozoite to 5 host cells for 1 hour. LDH was measured concurrently in supernatants to measure cell killing and graphed as the mean of three independent experimental values and s.e.m . **P < 0.001 (+EH) vs. (−EH) by Fisher’s LSD test. (**b**) HT-29 cells were loaded with the K^+^-sensitive fluorescent dye PBFI to measure intracellular K^+^ concentration ([K^+^]_I_) upon interaction with *E. histolytica.* Cells showed a significant reduction in [K^+^]_I_ after 30 minutes of contact with *E. histolytica.* The mean of 3 biological replicates for each experimental condition relative to the mean of untreated wild type cells is shown; error bars represent the s.e.m. (*P < 0.05; **P < 0.005; ***P < 0.001 calculated by two-tailed t test).

We monitored extracellular levels of K^+^ levels and LDH in host cells exposed to *E. histolytica* over an hour. We found that increased extracellular K^+^ preceded elevated LDH, suggesting that K^+^ efflux occurred prior to cell death (Fig. 6a). To further determine if *E. histolytica* induced efflux of intracellular K^+^, cells were loaded with the K^+^-sensitive fluorescent intravital dye PBFI. Changes in intracellular K^+^ concentrations in HT-29 cells during contact with *E. histolytica* were monitored over 30 minutes in M199 media (KCl = 5.33 mM^64^). Cells co-incubated with *E. histolytica* displayed a significant reduction in intracellular K^+^ after 30 minutes (Fig. 6b). The extracellular ionic concentrations of K^+^ in the supernatants of HT-29 cells were measured in parallel and an increase of 0.1 mM K^+^ in the presence of *E. histolytica* was detected after 30 minutes. No increase in extracellular K^+^ concentration was detected in the absence of *E. histolytica* or in *E. histolytica* alone (data not shown).

### Inflammasome activation

K^+^ efflux is a well-defined mechanism of inflammasome activation and pyroptotic cell death via caspase-1 activation in human cells^65^,^66^,^67^. *E. histolytica* has recently been reported to activate the host cell inflammasome in THP-1 macrophages^68^. Intestinal epithelial cells have also recently been shown to express a caspase-4 dependent IL-18 secreting inflammasome^69^,^70^. We hypothesized that *E. histolytica-*induced K^+^ efflux would trigger inflammasome activation in intestinal epithelial cells. *E. histolytica* did not induce secretion of IL-18 or IL-1β in HT-29 cells (Fig. S2). ATP treatment of LPS-primed HT-29 cells, a positive control for NLRP3 inflammasome activation, also failed to trigger IL-18 or IL-1β in HT-29 cells (data not shown). Specific inhibitors of caspase 1, 3, 4 and the pan-caspase inhibitor zVAD-FMK protected HT-29 IECs from amebic killing but without secretion of IL-18 or IL-1β caspase activation of a putative inflammasome in IECs remains undefined (Fig. S2).

**Figure 7:**
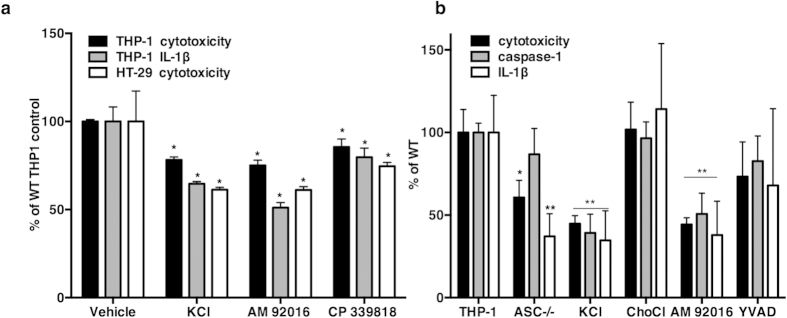
Inhibition of K^+^ channel activity blocked amebic cytotoxicity in HT-29 IECs and amebic cytotoxicity and IL-1β production in THP-1 macrophages. (**a**) IL-1β secretion was measured by ELISA in HT-29 cells and differentiated THP-1 macrophages exposed to *E. histolytica* for 180 minutes (1 trophozoite to 5 cells). HT-29 cells did not secrete a detectable level of IL-1β (data not shown). Inhibitors did not cause LDH release in the absence of *E. histolytica*. Experimental values were normalized and expressed as the percent of vehicle treated controls, the mean and s.e.m. of three biological replicates is shown. P values were calculated relative to untreated cells (*P < 0.05; **P ≤ 0.008) by two-tailed Fisher’s LSD test. (**b**) LDH, IL-1β and cleaved caspase-1 in cells treated with KCl (50 mM), ChoCl (50 mM) AM92016 (10 μK) and YVAD (10 μ). Each experimental condition was normalized and is expressed relative to untreated wild type cells; the mean and s.e.m. of three biological replicates is shown. P values were calculated relative to untreated cells (*P ≤ 0.02; **P ≤ 0.005) by Fisher’s LSD test.

The NLRP3 inflammasome is activated by multiple stimuli and K^+^ efflux is reported to be the common trigger for diverse stimuli^66^. We found that *E. histolytica* inflammasome activation in THP-1 macrophages required K^+^ efflux as increased extracellular K^+^ and specific K^+^ channel inhibitors (AM 92016 and CP 339818) blocked IL-1β secretion and amebic cytotoxicity in THP-1 cells (Fig. 7a). We tested the effect of caspase inhibitors on inflammasome activation and killing of THP-1 cells by *E. histolytica.* Caspase-1 inhibition blocked both cell killing and IL-1β secretion. Caspase-3 inhibition blocked cell killing but not IL-1β secretion, while caspase-4 inhibition had no effect. The pan-caspase inhibitor zVAD-FMK significantly inhibited IL-1β secretion and amebic cytotoxicity in THP-1 cells (Fig. S2). Thus both caspase-1 and K^+^ efflux were required for IL-1β secretion in response to *E. histolytica* by THP-1 macrophages.

ASC (apoptosis-associated speck-like protein containing a C-terminal caspase recruitment domain) is a key adaptor molecule between pathogen-sensing NOD-leucine-rich repeat (NLR) proteins, and pro-caspase-1. ASC mediates oligomerization into the inflammasome complex, which leads to auto-activation of pro-caspase-1 and the secretion of IL-1β and IL-18^71^. To further investigate inflammasome activation we tested the susceptibility of ASC-deficient THP-1 cells to *E. histolytica* killing. ASC-deficient THP-1 cells were significantly more resistant to amebic killing (39.3 .7± 5.9% reduction in killing vs. wild type THP-1 cells, *P* = 0.02). ASC-deficient cells secreted significantly less IL-1β but only moderately less processed caspase-1 (Fig. 7b). Caspase-1 secretion in response to *E. histolytica* required K^+^ efflux and was inhibited by excess K^+^ and the K^+^ channel inhibitor AM 92016. It is of note that excess K^+^ was more effective than the caspase-1 inhibitor YVAD, for blocking caspase-1 activation, IL-1β secretion and cell killing by *E. histolytica* (Fig. 7b).

## Discussion

Our goal was to identify novel host factors in amebic cytotoxicity by screening a genome-wide RNAi library of human cells with live parasites. The screen identified genes and pathways previously implicated in amebic cytotoxicity as well as many genes that have not been previously explored in amebiasis. Whole-genome pooled libraries are an efficient and powerful method of identifying novel host genes. The TRC library was made with shRNA constructs shown to yield stable, long term gene knockdown without activating host interferon responses^72^. However, whole-genome shRNA screens suffer from several inherent limitations including off-target effects and incomplete knockdown of target genes. Thus validation of hits from the primary screen was critical. We selected a subset of hits identified in the primary pooled screen and validated these in a secondary screen using different RNAi technology and an independent assay for amebic cytotoxicity. Approximately 70% of the genes identified in the primary screen reduced cytotoxicity in the secondary screen, although some only marginally. Our primary screen was designed with multiple steps over several weeks, thus even small increases in resistance may have been advantageous as the population underwent selection. Interestingly, several knockdowns in the secondary screen significantly increased amebic cytotoxicity. This may be due to false-positive hits in the primary screen, RNAi induction of target gene transcription^73^ and/or the difference in experimental design between the primary and secondary screen.

We decided to pursue K^+^ channels as novel mediators of host cell killing by *E. histolytica*. We found that blockade of K^+^ efflux, genetically (Fig. 2), pharmacologically (Figs 3 and 5b), and electromotively (Fig. 5a) inhibited *E. histolytica* cytotoxicity. We also showed that *E. histolytica* activated human K^+^ channels (Fig. 5d) and decreased intracellular K^+^ concentrations in host cells while simultaneously raising extracellular K^+^ concentrations (Fig. 6), indicating that parasites directly activate of K^+^ efflux. Finally, we found that *E. histolytica-*induced K^+^ efflux was required for inflammasome activation and cell death in human THP-1 macrophages. The importance of K^+^ ion transport for *E. histolytica* cytotoxicity and inflammation is consistent with the physiologic diarrheal symptoms of amebic colitis.

*E. histolytica* secretes analogs of host serotonin and PGE2, these molecules and other virulence factors may be responsible for parasite activation of ion transport. Enterotoxic bacteria including *Vibrio cholera*^74^ and *Escherichia coli*^75^ secrete toxins that manipulate ion-transport in the intestinal epithelium via activation of adenylate cyclase, leading to increased intracellular cAMP, leading to Cl^−^ secretion via Cftr and severe diarrhea. Analogously, previous work has defined a role for Na^+^, Cl^−^ and Ca^2+^ ion transport in amebic cytotoxicity. The slow Na^+^-Ca^2+^ channel blockers verapamil and bepridil^76^ and inhibitors of Ca^2+^ flux have been shown to block amebic cytotoxicity *in vitro*^33^,^34^. In addition, lysates of *E. histolytica* inhibited colonic Na^+^ and Cl^−^ absorption while stimulating luminal Cl^−^ secretion via a Ca^2+^-dependent response activated by amebic serotonin^24^ and a Ca^2+^-independent response activated by increased cellular cAMP^23^. Amebic lysates contain many toxic insults, which may indirectly activate ion channels or permeabilize cell membranes. Our data adds to previous studies by clearly demonstrating a direct effect of live *E. histolytica* parasites on host ion transport in multiple cell types and by the novel identification of the importance of K^+^ channels in amebic pathogenesis.

**Figure 8:**
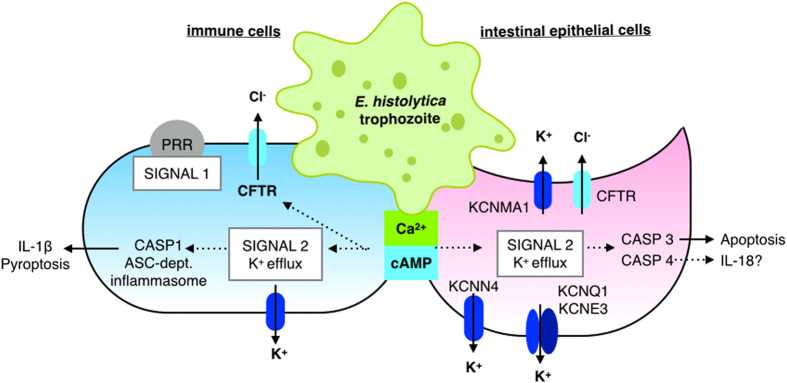
Model for *E. histolytica* activation of K^+^ channels in host cell killing. In both intestinal epithelial and immune cells, *E. histolytica* triggers increased intracellular Ca2^+^ and cAMP. In an intestinal epithelial cell (purple cell on the right), Cl^−^ efflux is mediated by apical Cftr, while K^+^ efflux occurs at both apical and basolateral surfaces. Increased intracellular Ca2^+^ activates large conductance K^+^ channels in the apical and basolateral membranes. Cl^−^ efflux may be activated directly by increased intracellular Ca2^+^ and/or cAMP or may occur secondarily to K^+^ efflux to regulate cellular charge polarization. As K^+^ and Cl^−^ efflux occurs, water and intracellular ion concentrations fall which causes cells to shrink. Cell shrinkage and decreased intracellular K^+^ trigger caspase activation. In intestinal epithelial cells it appears that caspase-3 is activated leading to apoptotic death. In macrophages (blue cell on the left), decreased cytosolic K^+^ concentration mediated caspase-1 activation leading to inflammasome activation, IL-1β secretion and pyroptotic cell death.

At the intestinal epithelium Cl^−^ efflux is mediated by the apical Cftr (identified in our RNAi screen), while K^+^ efflux occurs at the apical and basolateral surface^100^,^101^,^102^. Intestinal cells are extremely sensitive to intracellular Ca^2+^ and cAMP concentration which activate K^+^ channels in luminal and basolateral membranes^51^,^52^,^62^,^69^,^70^. A model of ion transport induced by *E. histolytica* via increased intracellular Ca^2+^ and cAMP levels is shown in Fig. 8. It remains to be determined if K^+^ and Cl^−^ efflux are each triggered directly or if one occurs secondarily to balance charge across the membrane. In the event of K^+^ and Cl^−^ efflux, intracellular ion concentrations fall, triggering water secretion and cell shrinkage leading to apoptotic volume decrease death (AVD). Cytosolic K^+^ concentration regulates caspase activation^77^,^78^ and increased extracellular K^+^ and inhibitors of Ca^2+^-activated K^+^ channels blocked intrinsic and extrinsic apoptotic pathways^79^. Low intracellular K^+^ concentrations activated caspase-1 and pyroptotic cell death^65^,^66^,^67^. We found that caspase-1 activation was blocked by high extracellular K^+^ and by specific inhibitors of voltage-gated K^+^ channels in macrophages (Fig. 7). Caspase inhibitors also reduced cell death in IECS in the apparent absence of the inflammasome. These data led us to hypothesize that decreased cytosolic K^+^ concentration is the common biochemical signal that mediates rapid activation of different caspases in multiple cell types by *E. histolytica*.

ASC was required for pro-caspase-1 autoproteolysis and IL-1β secretion by the NLRC4, NLRP3 and AIM2 inflammasomes but not the NLRP1b inflammasome^80^. Pro-caspase-1 can also be non-canonically activated by caspase-11 and appears to be involved an activation cascade during the inflammatory response^81^,^82^. The finding that ASC deletion impaired cell killing and IL-1β secretion, but not caspase-1 activation may indicate that inflammasome activation by *E. histolytica* proceeds through non-canonical K^+^-dependent pathways, potentially via multiple NLRs.

K^+^ channels are the most complex class of ion channels in both structure and function, with diverse expression and function throughout the body. Our analysis found that K^+^ channels are highly expressed and regulated in the human colon during *E. histolytica* infection (Fig. 4). Another important consideration in our interpretation is that the majority of these studies were done in immortalized cultured human cells. The initial screening was performed in UMUC3 epithelial cells, while validation was performed in UMUC3 cells, HT-29 IECs, and THP-1 macrophages. We found that K^+^ efflux is a new and critical host mediator of amebic cytotoxicity across multiple cell types.

K^+^ channel inhibitors have different effective concentrations across channel subtypes and tend to be less specific with increasing concentration. Several of the ion channel inhibitors we tested blocked amebic cytotoxicity at concentrations above reported IC_50_ values likely due to broader inhibition of ion flux at these concentrations. Several inhibitors had differential efficacy in HT-29 IECs versus THP-1 macrophages (Figs 5 and 7a). This may be explained by differential expression of Kcn genes in these cell types. Paxilline was most effective inhibitor of killing of IECs by *E. histolytica*. Paxilline specifically inhibits Kcnma1, an apical K^+^ channel localized to goblet cells in the colon^49^. Apical Kcnma1 is activated by Ca^2+^^83^. Notably Kcnma1 channels were localized more deeply in crypts in human ulcerative colitis^84^. Kcnma1 overexpression enhanced K^+^ secretion in experimental colitis^85^ while deletion abolished luminal colonic K^+^ secretion in mice^86^,^87^. Kcnma1 is also expressed by macrophages and regulated IL-6^88^, supporting our finding that paxilline was an effective inhibitor of amebic killing of THP-1 macrophages (Fig. 5b). These studies in combination with our data suggest that Kcnma1 may be a critical regulator of inflammation in intestinal epithelial cells and immune cells.

In contrast to intestinal epithelial cells, AM 92016, a blocker of delayed rectifier K^+^ channels and CP 339818, a blocker of voltage-gated K^+^ channels were most effective for preventing amebic killing of THP-1 macrophages. Both inhibitors block KcnA3, which was identified in the RNAi screen. Kcna3 is expressed in lymphocytes^61^ and epithelia^89^ and upregulation of Kcna3 was associated with Crohn’s disease^90^. This finding is consistent with our data that K^+^ channels are required for inflammasome activation in macrophages (Fig. 7a,b) and suggests that Kcna3 could be a specific anti-inflammatory drug target in multiple tissues.

Several key questions remain about the role of ion flux induced during *E. histolytica* pathogenesis. The specific K^+^ channels that are activated in human colonic epithelial and infiltrating immune cells remain unknown. Our data suggest that *E. histolytica* activates K^+^ channels via increased cytosolic Ca^2+^ and possibly cAMP (Fig. 5b). Future directions will be to determine the mechanism of cell death induced by K^+^ efflux in different cell types. Our current hypothesis is that decreased intracellular K^+^ concentration mediates caspase activation across diverse cells types. Activation of caspase-3^91^ and apoptotic cell death may be advantageous to minimize inflammation at the intestinal epithelium during colonization by *E. histolytica*. Activation of caspase-1 in macrophages during invasive disease may lead to enhanced secretion of pro-inflammatory cytokines. We propose a model where decreased intracellular K^+^ concentration is a common signal to activate host caspases in diverse cell types^71^. It will be critical to define how K^+^ efflux is involved in distinct forms of cell death during amebic infection. Further, K^+^ channel inhibitors may have clinical promise for blocking both parasite virulence and host inflammation at the intestinal epithelium.

The unexpected finding that K^+^ efflux triggers cell killing, and activation of inflammatory cascades by the diarrheal pathogen *E. histolytica* is an important advance in the understanding of amebic pathogenesis. Host ion transport is a critically important area for future investigation as it also is the primary mechanistic cause of amebic diarrhea. Our work additionally indicates that K^+^ efflux activates inflammatory cascades in host immune cells. Therefore, specific inhibition of host ion efflux, in particular K^+^ efflux, may represent a novel, host-directed therapeutic intervention to block the damaging secretory and inflammatory responses elicited by diarrheal pathogens.

## Methods

### Cell culture

UMUC3 human bladder epithelial cells were maintained in Minimum Essential Medium (MEM) with 2 mM L-glutamine and 10% heat-inactivated FBS. HT-29 cells were maintained in McCoy’s 5a with 10% heat-inactivated FBS. *E. histolytica* strain HM1:IMSS trophozoites were grown at 37 °C in TYI-S-33 medium supplemented with penicillin (100 U/mL) and streptomycin sulfate (100 μg/mL). All co-culture of human cells with *E. histolytica* trophozoites was done in M199 supplemented with 5.7 mM cysteine, 0.5% BSA, and 25 mM HEPES (pH 6.8). The UMUC3 cells were infected by lentiviral transduction with the RNAi Consortium 1.0 shRNA library or an empty vector control as described by Guin *et al.*^92^. Transduced cells were maintained under puromycin selection at 2 μg/mL.

### Library Screening

The UMUC3 TRC 1.0 library was distributed into eight tissue culture treated T75 flasks to a final concentration of 2.0 × 10^5^ cells/mL. UMUC3 cells transduced with an empty vector shRNA were seeded equivalently into two T75 flasks and allowed to adhere for three hours. The media was removed and *E. histolytica* trophozoites were added at a concentration of 2.0 × 10^4^ trophozoites/mL (a ratio of 1 parasite to 5 host cells) in M199S. Cells were incubated with *E. histolytica* for three hours and mixed by gentle rocking every 15 minutes. After selection, the host cell monolayers were washed once with 5 mL MEM. Washes were centrifuged at 1000 × g for 5 minutes to recover detached cells which were resuspended in complete MEM and transferred back to the surviving monolayers. The monolayers were grown to ~90% confluence. Trophozoites did not survive in MEM with puromycin. For re-screening, monolayers were trypsinized and plated at 2 × 10^5^ cells/mL.

### Next generation sequencing to identify candidate susceptibility genes

Genomic DNA was isolated from the pools of selected cells using the GenElute mammalian DNA kit (Sigma Aldrich). Sequencing libraries were created from genomic DNA using primers designed to specifically amplify shRNA inserts. The flow cell was built on an Illumina cBot cluster generation station using GA Compatible (cBOT) cluster generation kits (Illumina). Sequencing was carried out on an Illumina GaIIx sequencer using a 36-cycle single-end run with a 36-Cycle Sequencing Kit v4 (Illumina). Raw sequencing reads were aligned to the expected computational target sequences from the TRCN clones. Reads containing a full-length perfect match alignment to a clone target sequence were flagged as hits. Hits with fewer than ten sequence reads were omitted from the analysis.

### Secondary validation

55 genes identified in the original screen were tested in a secondary screen using endoribonuclease-prepared siRNAs (esiRNA) (Sigma-Aldrich). esiRNA transfection conditions were optimized with an esiRNA targeted to firefly luciferase (FLUC) in UMUC3 cells stably expressing FLUC. Knockdown efficiency was confirmed by qRT-PCR for 5 genes and esiRNA knockdown yielded >75% knockdown for all genes. Each transfection was done in triplicate in a 96-well plate with 500 cells, 24 ng/well esiRNA and 0.2 μL oligofectamine RNAi max (Life Technologies)/well. Media was replaced after 24 hours; cytotoxicity assays were done 48 hours post-transfection. Cytotoxicity was normalized to firefly luciferase (FLUC) transfected controls.

### Amebic cytotoxicity assays

*E. histolytica* trophozoites were added to monolayers in M199S at the indicated concentrations and incubated at 37 °C with 5% CO_2_. At each time point plates were centrifuged at 500 × g for 5 minutes and 50 μL of supernatant was transferred to a black 96-well plate. Lactate dehydrogenase (LDH) levels in the supernatant were measured with the CytoTox-ONE Homogeneous Membrane Integrity assay (Promega) as directed. Briefly, 50 μL reconstituted CyTox-ONE reagent was added to each well. Plates were incubated for 10 minutes at 22 °C and 50 μL stop solution was added to each well and fluorescence was measured (560 nm excitation/590 nm emission). Percent cytotoxicity was calculated as: *[(LDH release* + *E. histolytica)* − *(LDH - E. histolytica)]*/*[maximum LDH release].* Maximum LDH release was determined by the addition of 0.2% Triton-X to cells alone.

### Bioinformatics

The DAVID Bioinformatics resource 6.7 was used to generate functional annotation gene lists for candidate genes^93^,^94^,^95^,^96^. The starting lentiplex shRNA input library of 16,058 gene knockdowns was used as the background list. 278 of the 281 candidate susceptibility genes mapped to a DAVID ID. Functional annotation clustering by biological process (BP), molecular function (MF) and cellular component (CC) was done to identify enriched gene ontology terms. BP describes genes in a recognized series of events with a defined beginning and end. MF describes the cellular functions of a gene product. CC describes the subcellular locations of proteins. EASE scores are modified Fisher exact P-value for gene-enrichment analysis (>0.05 was our cutoff for significance)^97^. The significantly enriched terms listed in Fig. 1d were manually curated to remove parental and redundant terms. The complete analysis is provided in Table S3. Additional functional annotation for enriched keywords done with the UniProt Knowledgebase, a comprehensive database of functional protein information.

### Inhibition of ion channels

5 × 10^4^ cells/well were plated in 96 well plates and incubated overnight. THP-1 cells were treated with 1 ng/mL PMA to induce differentiation. The day of the experiment media was changed to M199S. Cells were treated with inhibitors at the indicated concentrations in M199S for 30 minutes prior to the addition of *E. histolytica* (5 × 10^3^ trophozoites/well). For washout experiments, medium containing the inhibitor was removed from the cells and monolayers were washed twice in warm M199S prior to the addition of *E. histolytica.* Cytotoxicity was measured by LDH release as described above after 60 minutes.

IC_50_ concentrations were determined with GraphPad Prism software (version 6.0e). Cellular LDH values were normalized to 100% and 0% based on external controls where the mean of vehicle-treated cells in the presence of *E. histolytica* was set to 100% and the mean of vehicle-treated cells in the absence of *E. histolytica* was set to 0%. DMSO at the same concentration used for compound addition served as the negative vehicle control. IC_50_ inhibition curves were fitted using the normalized least squares (ordinary) fit method. In cases where inhibition curves plateau above the control values (0%) the IC_50_ defined the middle of the curve and the concentration of which yielded the maximum inhibitory concentration (IC_max_) is shown. Several inhibitors resulted in cellular toxicity at higher concentrations in the absence of *E. histolytica*. In these instances, the points are shown for reference, but values were excluded from IC_50_ determinations.

### Gene expression analysis

The methods are described in the original report of this analysis^37^. Colonic biopsy samples were obtained from 8 subjects with acute *E. histolytica* colitis, and again 60 days later during convalescence. Gene expression in the human colon during acute and convalescent *E. histolytica* disease was evaluated by microarray (data are deposited under GEO accession GSE23750)^37^.

### K^+^ channel activation assays

The FluxOR™ potassium channel assay (Life Technologies) was performed as previously described^98^. Briefly, 40 μL of loading buffer with dye was added to cells in 96 well plates for 60 min at room temperature and then removed manually. Cells were washed once with dye-free assay buffer before the addition of 80 μL assay buffer containing inhibitors to each well. Cells were incubated at room temperature for 30 min. 20 μL of *E. histolytica* trophozoites resuspended in stimulation buffer (chloride-free buffer with 10 mM free Tl^+^) was added to each well resulting in 2 mM free Tl^+^ and 1 trophozoite to 5 host. Controls of cells without *E. histolytica* and inhibitors were tested in parallel. Fluorescence was measured every 40 seconds for 12 minutes following stimulation.

Baseline fluorescence value for each well was set to the mean of 3 readings before addition of stimulus buffer. Each F value was normalized to the mean initial baseline value. The effect of inhibitors on K^+^ channel activation by *E. histolytica* was quantified by the difference in the area under the curve (AUC) of the fluorescence over 12 minutes relative to untreated cells. We tested inhibitors at a single concentration (KCl and ChoCl = 25 mM, 293B, CLO and PAX = 10 μM). DMSO was the vehicle control. P values were calculated relative to untreated cells by Fisher’s least significant difference (LSD) test (*P < 0.001).

### PBFI intracellular K^+^ measurements

HT-29 cells were seeded in 96-well plates at 5 × 10^5^ cells/well and allowed to settle, after which the medium was changed to M199S. Cells were loaded with the acetoxymethyl ester of PBFI (PBFI-AM) (Life Technologies) at 5 μM at room temperature for 60 minutes followed by two washes with M199S. Valinomycin was used as a positive control. Fluorescence was measured at excitation 340 nm/500 nm emission and 380 nm excitation/500 nm emission. PFBI fluorescence at 340 nm excitation/500 nm emission is K^+^-dependent while 380 nm excitation/500 nm emission is K^+^-independent; hence the 340/380 excitation ratio controls for variations in dye concentration and photobleaching. After a five-minute initial period of stabilization, cells were treated as indicated.

### Measurement of extracellular K^+^

1 × 10^6^ HT-29 cells/mL were plated in 6 well plates and grown overnight. The following day, 1 well was used to obtain a cell count. For the remaining wells, medium was replaced with M199S containing *E. histolytica*. 100 μL samples were taken at the time points indicated and centrifuged at 1000 × g. Supernatants were transferred to a new microfuge tube and immediately stored at −20 °C. The experiment was performed in triplicate. K^+^ concentrations in sample supernatants were measured at the University of Virginia Health system clinical chemistry labs. The coefficient of variance of the assay at this level was 1%.

### Inflammasome activation

HT-29 or THP-1 cells were seeded in 6 well plates the day before the experiment in cell culture media supplemented with 5 ng/mL PMA the day before the experiment. The next day, medium was replaced with serum free RPMI 1640 prior to addition of inhibitors. All inhibitors with the exception of KCl were washed out prior to addition of *E. histolytica* trophozoites in serum free RPMI1640. After a 3-hour incubation, the plates were centrifuged and supernatants were analyzed separately for IL-1β secretion and caspase-1 secretion by ELISA specific to the p20 subunit of caspase-1 (R&D biosystems) and cell lysis by LDH release as described above. The processing of IL-1β in supernatants was verified by immunoblot.

### Statistical analysis

Statistical significance was calculated with GraphPad Prism software (version 6.0e). The statistical tests used are noted in the figure legends. Boinformatics analysis was performed with DAVID 6.7 tools to calculate Fisher’s exact P value, ≤0.05 was considered significant.

## Additional Information

**How to cite this article**: Marie, C. *et al.* A whole-genome RNAi screen uncovers a novel role for human potassium channels in cell killing by the parasite *Entamoeba histolytica*. *Sci. Rep.* **5**, 13613; doi: 10.1038/srep13613 (2015).

## Supplementary Material

Supplementary Information

Supplementary Tables 1-4

## Figures and Tables

**Table 1:** Sequencing results of selected libraries at round 6, 8 and 9 of selection.

**Pool**	TRCN Clones	Target Genes	Genes targetedby >1 shRNA	% of input library(clones, genes)
6	5320	4314	760	6.6, 26.9
8	410	395	15	0.5, 2.5
9	284	281*	3	0.35, 1.7

^*^Candidate gene list for subsequent analysis.

**Table 2:** Overrepresented functional categories in candidate susceptibility genes (Pool 9).

**Category**	**Term**	**Count**	**%**	**P-Value**	**Enrichment**
BP	cell death	25	9	0.00037	2.20
MF	transcription activator activity	17	6	0.00052	2.70
BP	transmembrane transport	21	8	0.00062	2.40
MF	ion transmembrane transporter activity	24	9	0.00065	2.20
MF	substrate-specific transmembrane transporter activity	26	9	0.00076	2.10
MF	transmembrane transporter activity	27	10	0.00130	2.00
CC	intracellular organelle	155	56	0.00260	1.20
MF	gated channel activity	12	4	0.00770	2.50
MF	binding	204	74	0.00850	1.10
MF	cation transmembrane transporter activity	17	6	0.01100	2.00
MF	anion binding	6	2	0.01300	4.30
BP	transport	56	20	0.01400	1.40
MF	transferase activity	39	14	0.01400	1.50
MF	ion channel activity	13	5	0.01500	2.20
CC	integral to plasma membrane	28	10	0.01700	1.60
MF	substrate specific channel activity	13	5	0.01800	2.20
CC	apical part of cell	8	3	0.02100	3.00
CC	plasma membrane part	45	16	0.02200	1.40
CC	ion channel complex	8	3	0.03300	2.60

BP (Biological Process), MF(Molecular Function), CC (Cellular Component).

**Table 3:** K^+^ channel candidate susceptibility genes in pool 9.

**Gene name**	**Description**	**esiRNA**	**EXP**	**REG**	**Evidence score**
Kcna3	Voltage-gated, shaker-related	84% P <0.0001	++++	−0.07	5
Kcnb2	Voltage-gated delayed rectifier, Shab-related	84% P = 0.03	++	0.07	4
Kcnip4	Voltage-gated interacting protein, binds Ca^2+^	124% P = 0.01	+	−0.22	1
Kcnj3	K^+^ inwardly-rectifying channel	83%	+++	−0.07	5
Slc24a3	Solute carrier, Ca^2+^/Na^+^/K^+^ exchanger	107%	++++	−0.15	2

esiRNA: mean % killing relative to FLUC control. P calculated relative to FLUC control by 2-tailed t test.

EXP: level of colonic expression (+) 0–25%, (++) 25–50%, (+++) 50–75%, (++++) 75–100%.

REG: mean difference in gene expression between day 1 of acute amebiasis and day 60 (recovery) (n = 8, matched samples).

**Table 4:** Effect of channel inhibitors on amebic killing of UMUC3 cells.

**Inhibitor**	**No washout**		**Washout**	
	IC_50_(μm)	IC_max_(% killing)	IC_50_(μm)	IC_max_(% killing)
ibutilide (IBU)	4.4	47 (0%)	4.7	47 (0%)
5-nitro-2-(3-phenylpropylamino) benzoic acid (NPPB)	4.6	50 (6%)	14.9	94 (10%)
benzamil (BNZ)	8.2	50 (1%)	4.4	47 (7%)
tetraethylammonium chloride (TEA)	45.4	234 (0%)	37.2	234 (11%)
diltazem (DIL)	25.9	50 (12%)	57.6	750 (25%)
4-aminopyrimadine (4-AP)	1366	188 (44%)	807	188 (44%)
procaine (PRO)	1571	375 (32%)	35.8	4.7(9%)
quinine (QUI)	712	47 (51%)	653	47 (46%)

IC_max_- concentration with maximum inhibition of amebic cytotoxicity.

The % killing at the IC_max_ relative to untreated cells is shown in parenthesis.

**Table 5:** Effect of specific channel inhibitors on amebic killing intestinal epithelial cells (HT-29) and macrophages (THP-1).

**Inhibitor**	**HT-29**			**THP-1**		
	**IC**_**50**_	**95% CI**	**IC**_**max**_	**IC**_**50**_	**95% CI**	**IC**_**max**_
KCl	6.8 mM	5.2–8.9	50 mM (3.6%)	11.9 mM	8.4–16.8	12.5 mM (33.7%)
K_2_S0_4_	9.1 mM	5.2–16.1	12.5 mM (30.7%)	13.8 mM	6.2–30.8	25 mM (32.6%)
ChoCl	52.2 mM	34.2–79.8	50 mM (36.4%)	29.2 mM	16.3–52.3	12.5 mM (44.9%)
NaCl	21.8 mM	13.3–35.7	50 mM (11.6%)	58.9 mM	30.6–113.2	50 mM (41.5%)
Clotrimazole (CLO)	5.9 μM	4.0–8.8	6.25 μM (24.3%)	13.9 μM	7.4–26.0	6.25 μM (41.3%)
Paxilline (PAX)	4.9 μM	3.6–6.6	25 μM (16.0%)	6.2 μM	4.4–8.6	50 μM (14.4%)
Chromanol 293 (293B)	6.9 μM	4.2–11.3	25 μM (42.6%)	7.8 μM	5.1–12.1	25 μM (37.7%)
AM 92016	7.3 μM	3.9–13.8	12.5 μM (39.1%)	4.1 μM	2.5–6.7	12.5 μM (9.3%)
CP 339818	9.0 μM	5.2–15.4	50 μM (35.8%)	4.7 μM	3.7–5.9	50 μM (13.5%)
TEA	7.8 μM	5.0–12.3	12.5 μM (36.1%)	20.5 μM	11.5–36.4	12.5 μM (53.8%)

IC_max_- concentration with maximum inhibition of amebic cytotoxicity. The % killing at the IC_max_ relative to untreated cells is shown in parenthesis (untreated-IC_max_ treated).

• K_2_SO_4_ concentration is shown as K^+^ ion concentration (twice the concentration of K_2_SO_4_).
