# Supplementary material for: A whole-genome RNAi screen uncovers a novel role for human potassium channels in cell killing by the parasite Entamoeba histolytica
Source: Sci Rep. 2015 Sep 8;5:13613. doi: 10.1038/srep13613 (PMC4561901; doi:10.1038/srep13613)
Supplement: Supplementary Information [file srep13613-s1.doc]

**Supplemental Figures and Legends**

**Title:** A whole-genome RNAi screen uncovers a novel role for human potassium channels in cell killing by the parasite *Entamoeba histolytica*

**Authors:** Chelsea Marie^1^, Hans P. Verkerke^1^, Dan Theodorescu^2-4^, William A. Petri, Jr.^1^


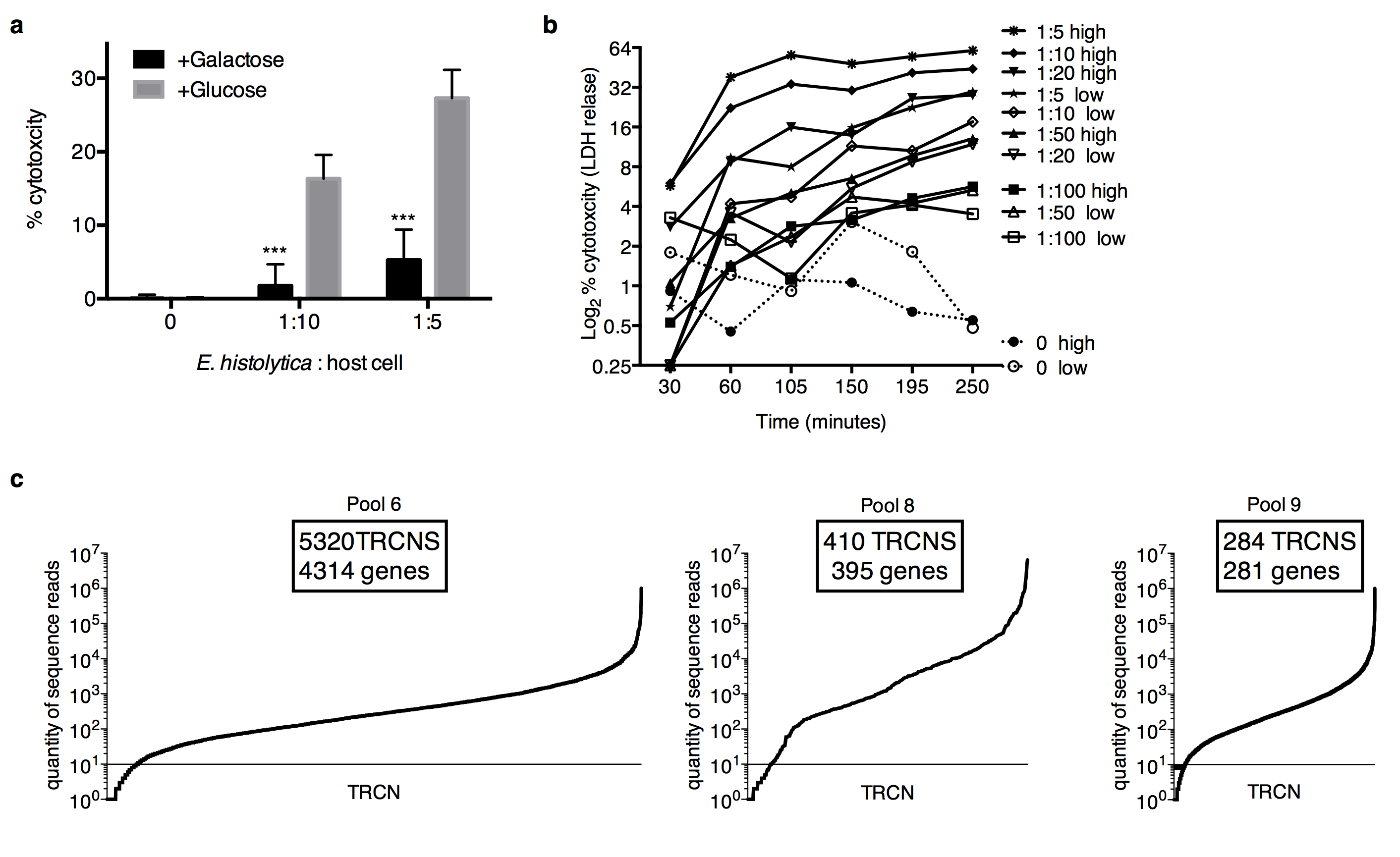


**FIGURE S1. Validation and optimization of a whole genome RNAi screen to identify host factors important in amebic cytotoxicity.** (a) UMUC3 cells were susceptible to amebic cytotoxicity and killing was inhibited by 100 mM galactose at ratios of 1:5 and 1:10 parasite to UMUC3 cells. 100 mM glucose was tested as an osmotic control. ***P=0.0005. (b) Survival of UMUC3 cells at high (5 x 10^5^ cells/ml) and low density (1 x 10^5^ cells/ml) plating. UMUC3 cells at a low density were less susceptible to amebic killing at the same parasite to host cell ratio than were host cells plated at a high density. The lower density was used at a 1:5 ratio of parasites to host cells to minimize potential bystander effects on adjacent clones during *E. histolytica* killing. (c) Pools of resistant clones were sequenced to identify the shRNA construct expressed in each UMUC3 cell present in a selected pool. Sequence abundance of each clone (TRCN ID) was plotted on a logarithmic scale. shRNA sequences were normally distributed in the selected libraries. Clones with fewer than 10 sequencing read were excluded from the analysis.


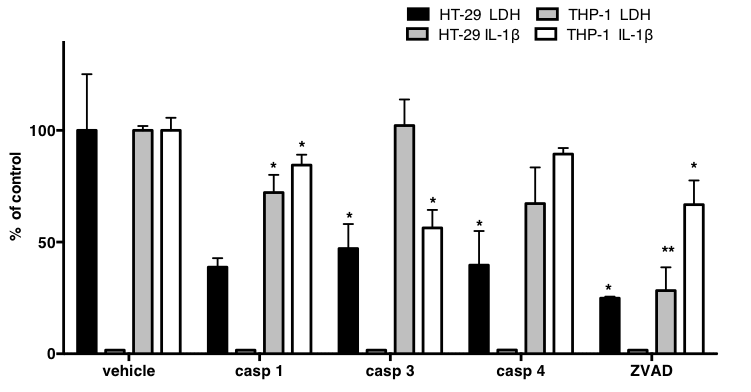


**FIGURE S2**. **Inhibition of host caspases blocked amebic cytotoxicity in HT-29 IECs and amebic cytotoxicity and IL-1β production in macrophages***.* IL-1β secretion by THP-1 macrophages exposed to *E. histolytica* for 180 minutes. Cytotoxicity was measured by LDH release and inhibition of cytotoxicity was calculated relative to untreated controls. The mean of triplicate experimental values is shown and error bars show the s.e.m. P-values were calculated relative to untreated cells by two-tailed student’s t-test (*P < 0.05; **P < 0.005).
